# Beliefs and misconceptions about contraception and condom use among adolescents in south-east Nigeria

**DOI:** 10.1186/s12978-020-01062-y

**Published:** 2021-01-06

**Authors:** Chinyere Ojiugo Mbachu, Ifunanya Clara Agu, Chinonso Obayi, Irene Eze, Nkoli Ezumah, Obinna Onwujekwe

**Affiliations:** 1 Health Policy Research Group, University of Nigeria Enugu, Nsukka, Nigeria; 2 Department of Community Medicine, University of Nigeria Enugu Campus, Nsukka, Nigeria; 3 Department of Health Administration and Management, University of Nigeria Enugu Campus, Nsukka, Nigeria; 4 Department of Community Medicine, Ebonyi State University Abakaliki, Abakaliki, Nigeria

**Keywords:** Misconceptions, Contraceptives, Condom, Adolescent, Sexual behaviours

## Abstract

**Background:**

Misconceptions about the usefulness of condoms and other contraceptives still expose many unmarried adolescents to the risk of unwanted teenage pregnancies and sexually-transmitted infections (STIs). This study explored beliefs and misconceptions about condoms and other contraceptives among adolescents in Ebonyi state, south-east Nigeria.

**Method:**

A qualitative study was undertaken in six local government areas in Ebonyi state, southeast Nigeria. Data were collected within a period of one month from in and out-of-school adolescents aged 13–18 years using twelve focus group discussions (FGD). The data were analyzed using the thematic framework approach.

**Result:**

Majority of the adolescents were knowledgeable about methods of contraception, how they are used and their modes of action. They were also knowledgeable about the dual effects of condoms in prevention of pregnancy and STIs. However, some misconceptions that were expressed by some adolescents were that pregnancy could be prevented by the use of (i) hard drugs, (ii) laxatives, (iii) white chlorine, and (iv) boiled alcoholic beverages. Condoms were described by some adolescent boys as reusable. Condoms were also perceived by some adolescents to reduce sexual pleasure, and this opinion was mostly held by boys. Coitus interruptus (withdrawal method) was therefore considered more preferable than condoms for prevention of pregnancy.

**Conclusion:**

Although majority adolescents have knowledge about contraception and condom use, some misconceptions still persist. These misconceptions put many adolescents at increased risk for pregnancy and STIs which are detrimental to their health and wellbeing. Concerted efforts should be made through educational and behaviour change interventions in schools and within communities to debunk persisting misconceptions about contraception including the use of condom, and properly educate adolescents on safe sex practices.

**Plain English summary:**

Adolescents engage in unprotected sexual intercourse and other risky sexual behaviours because of some mistaken beliefs and wrong impressions about how to prevent unwanted pregnancy. These risky sexual behaviours predispose adolescents to sexually transmitted infections, unsafe abortion and other reproductive health problems.

In this qualitative study, we explored some of these mistaken beliefs about condoms and other methods of preventing pregnancy. During focus group discussions, adolescents identified modern contraceptive methods, and described their modes of action and how they are used. They also discussed their contraceptive preferences and perceived effects of condoms on sexual pleasure.

Although some of these adolescents were able to correctly mention various types of contraceptives and their modes of action, there were numerous wrong impressions. Hard drugs, laxatives, white chlorine and boiled alcoholic beverage were listed as emergency contraceptive methods. Emergency pills were perceived to work by flushing away spermatozoa from a girl’s system after sexual intercourse. Male condoms were perceived to be potentially dangerous because they could break and enter into the body of the female sexual partner. Some adolescent boys had the notion that particular brands of male condoms could be washed and reused. Notions about condom use and sexual pleasure varied for girls and boys. Some adolescent girls perceived that condom use during sex increases sexual pleasure because of the assurance of being protected from STIs and pregnancy. Adolescent boys were of the opinion that condoms interfere with the pleasure of direct ‘flesh to flesh’ contact during sex. There was a general belief that contraceptive use in early age reduces fertility prospects for boys and girls.

Mistaken beliefs about methods of preventing pregnancy persist among adolescents, and this raises concerns about the quality of information they receive. Concerted efforts should be made to debunk these wrong beliefs and properly educate adolescents on safe sex practices.

## Background

Adolescence is a transitional period characterized mostly by significant changes in psychosocial, sexual development, and physical growth [1]. At this phase of human development, many tend to develop an increased interest in experiences and behaviors that are associated with adulthood, such as starting new relationship with the opposite sex, engaging in sexual activity and other risky behaviors [2].

Many adolescents tend to experiment and indulge in some risky sexual behaviours primarily due to the feeling of independence as well as poor access to adequate and appropriate information about sexual and reproductive health [3, 4]. Risky behaviours such as unprotected sexual activities among adolescents is a major public health concern. Literature reveals that majority of adolescents in Africa and Europe engage in sexual activity between the ages of 12–19 and most of them achieve sexual debut by 16 years [5, 6].

A considerable proportion of adolescents in sub-Saharan Africa engage in sexual activities in their middle teenage years within ages 14 and 15 [7]. Regional estimates from developing countries confirm that sexual debut in West African Countries mostly occurs during adolescent period [8]. This early sexual exposure predisposes many adolescents to unprotected sexual intercourse and increases the likelihood of unintended pregnancies, unsafe abortions and STIs.

Unplanned pregnancies among adolescents contributes significantly to maternal morbidity and mortality in this age group [9]. Millions of adolescent girls in Africa experience unwanted pregnancies annually and about 60% of these pregnancies are terminated through unsafe abortions [10, 11]. West Africa has the highest proportion of adolescent pregnancies when compared to other sub-regions in sub-Saharan Africa [8].

In Nigeria, the proportion of adolescents who have begun childbearing increases with age, from 2% at 15 years to 37% at 19 years [12]. Early pregnancies, both planned and unplanned, among adolescents constitutes a serious problem. Unplanned pregnancies among adolescents are not only detrimental to their health but also obstructs their socioeconomic development [9, 13]. Most victims of unplanned pregnancy are at a higher risk of educational disruption, early marriage with more children at shorter intervals, future unemployment leading to low income and poor living standards. Whereas, adolescents who do not experience unplanned pregnancy are more likely to further their education, become involved in the country’s workforce and have healthier life [9].

Poor knowledge and inconsistent/incorrect use of contraceptives contribute to high rate of unplanned pregnancies [14, 15]. Global, regional and national reports reveal that adolescents have the lowest contraceptive prevalence rate when disaggregated by age [16]. However, regional variations exist as 93% of adolescents in a developed country reported using modern contraceptives in their last sexual encounter compared to 48.7% of adolescents in a developing country [17, 18]. The latter figure corresponds with findings from studies in Nigeria that reported low contraceptive prevalence rates among young people [19, 20].

Misconceptions about contraception and contraceptive methods contribute to non-use of contraceptives among adolescents and young unmarried people. Some authors have reported misconceptions about side effects and health problems associated with contraceptive methods, as well as negative stereotypes about contraceptive users [21, 22]. Condom for instance, has been proven to effectively protect against pregnancy and sexually transmitted infections, including HIV [23]. However, there are misperceptions about how it is used and its effects on fertility and sexual pleasure, which have contributed to inconsistent use of condoms in sexual partnerships [22, 24].

This paper provides new and useful information from a study which explored in-depth the notions and misconceptions about contraception and condom use among adolescents in south-east Nigeria. Most research on misconceptions about contraception and condom use have focused more on married adults (couples) and unmarried youths [19, 25, 26]. Given that misinformation could be easily spread among young people especially adolescents, through social media, peers in school and community, it is important to explore the fallacies about contraceptives among adolescents. The knowledge that this paper provides will be invaluable in planning suitable interventions to address any knowledge gaps among adolescents.

## Methods

### Study design and area

The qualitative cross-sectional study was undertaken in six communities in Ebonyi State, which is located in south-east Nigeria. The state has an estimated population of 6,268,003 inhabitants, with over 40% under the age of 15 years [27]. The NDHS report shows that maternal mortality rate among girls aged 15–19 is 30.5%; and 9.6% of girls in this age group have already begun child bearing [28]. From each senatorial zone in Ebonyi state, we purposively selected 2 LGAs that were listed as having the highest rates of unwanted teenage pregnancies and abortions in the State.

### Study population and sampling

The study population comprised of in-school and out-of-school unmarried adolescents aged between 13 and 18. The decision to target unmarried adolescents aged 13–18 years was made following recommendations by key stakeholders in adolescent SRH in the study State, that majority of adolescents in the State achieve sexual debut at 13–15 years and that secondary school age ranges from 13–18 years. One community was purposively selected from each of the six local government areas (LGAs) to ensure geographic and geopolitical spread in terms of place of residence (urban and rural). In each community, adolescents were selected from secondary schools, trade centers and skill-acquisition centers. Adolescents who were not attending school and appeared to be well informed during our quantitative survey were purposively selected for interview. Also, in school adolescents were randomly selected from public secondary schools in each study community. They were all invited to participate in the focus group discussions (FGDs). Two FGDs were conducted in each community (giving a total of twelve) and participants ranged from 8 to 13 in number (see Table 1 below). A more comprehensive method of our study can be found in a published article [29].

**Table 1:** Socio-demographic distribution of focus group discussion (FGD) participants

Variables	Number of participants	Gender	
		Female	Male
Place of residence			
Urban	63	32	31
Rural	71	36	35
Age category			
13–14	32	20	12
15–16	53	27	26
17–18	49	21	28
Schooling			
In-school(JS1–SS3)	96	42	54
Out-of-school	34	26	12
Employment status for out-of-school			
Employed	29	17	12
Unemployed	9	9	–

^A total of 12 FGD; *JS* Junior secondary, *SS* Senior secondary^

^(Nature of employment: farming, trading, sales person and apprentice such as automobile mechanic, tailoring, tricycle and hair dressing)^

### Data collection

Data were collected using a pre-tested focus group discussion guide. The discussion guide was developed by a team of qualitative research experts. A stakeholder engagement meeting was held in Ebonyi state prior to the commencement of the research project and a relationship was established with the participants who attended meeting. The stakeholders were officials recruited from different government and non-governmental organizations which include; State ministry of health, State ministry of education, State ministry of information, State ministry of youth and sports development, State ministry of women affairs and social development, State house of assembly, State universal basic education board, State primary health care development agency, civil society organizations, religious and traditional leaders. Succeeding the meeting, community mobilization was carried out in each study community prior to data collection process. The data were collected for a period of one month, in the month of October, 2018. In each community, one FGD was conducted separately for boys and girls respectively.

The FGDs were conducted by experienced researchers in qualitative study, either in English or Igbo language (the local language), depending on participants’ preferences. All participants were informed of the study objectives before commencement of interviews. All interviews were audio recorded with the permission of participants. Hand-written notes were also taken.

### Data analysis

All audio files were transcribed in the language of interview verbatim, and translated to English language for those conducted in Igbo. Transcripts were edited in Microsoft Word and compared with hand-written notes to affirm accuracy. Each transcript was anonymised using unique codes developed by the research team and data were analysed using thematic framework approach.

The most comprehensive FGD transcript was selected and given to two independent researchers for detailed review and coding. Sub-themes relating to perception of contraception and contraceptive methods were generated and these formed the initial coding framework. The initial coding framework was then tested on two new transcripts, and subsequently refined to form the final framework. The final coding framework was then applied to all 12 transcripts, including the ones that were used for testing. The themes in the final framework are shown in Table 2. The findings were presented after the analysis to key stakeholders in Ebonyi state for validation of synthesized data through a workshop.

**Table 2:** Thematic coding framework for analyzing beliefs and perceptions about contraceptives

Major and minor codes/themes
Beliefs and misconceptions about types of contraceptives
Beliefs and misconceptions about modes of action of contraceptive methods
Beliefs and misconceptions about condom use Beliefs about reusability of condoms Beliefs about condom use and sexual pleasure
Beliefs and misconceptions about contraceptive use and future fertility

## Results

### Beliefs and misconceptions about types of contraceptives

Adolescents were asked to mention different method(s) of modern contraceptive(s), their uses and mode of action. Majority of adolescents were knowledgeable of various type of contraceptives and they correctly identified condoms, emergency and daily pills, implant, injection, withdrawal method, spermicide, intrauterine devices (IUD), sterilization and tubal ligation as modern methods of contraception. A few of them also mentioned total and periodic abstinence as contraceptive methods, and one person mentioned abortion as a method of contraception.

Nevertheless, there were numerous misconceptions about methods of contraception. Adolescents mentioned use of hard drugs, laxatives, white chlorine and boiled alcoholic beverage as emergency contraceptive methods. Some of the adolescents who had these misconceptions also described how these methods are used and their modes of action. For instance, with respect to boiled alcoholic beverages, their assumption was that absorption of alcoholic beverage through the intestine and into the bloodstream facilitates flushing of spermatozoa in urine from a girl who has just had sexual intercourse.

The following quotes highlight some correct notions and misconceptions about contraceptive methods. Some of these quotes also show the co-existence of correct and incorrect notions in the perception of the same individual.

“When we use condom is when a boy wants to have sexual intercourse with a girl, to prevent them from contracting any disease or for the girl not to become pregnant. Condom has advantages and disadvantages. If you don’t want to get pregnant after having sex you can take Andrew’s liver salt (laxative) and white chlorine. Some people take drugs also” (Female Adolescent—ADABF_R1).

“Condom will help the boy that when he wants to release sperm, he will release it inside the condom. For those who do not use condoms, when the boy releases immediately inside the girl’s body, she should take Andrew’s liver salt (laxative) and urinate immediately, it will neutralize the sperm in the girl’s body” (Male Adolescent—ADABM_R1).

“Some make use of hard drugs before sexual intercourse to prevent pregnancy. Pills are also used by the girls after having sexual intercourse” (Male Adolescent—ADAFM_R7).

“Some girls prevent pregnancy by using small stout (alcoholic beverage). They boil the small stout and drink it hot, and it will flush away the pregnancy. The hot small stout will wash away the sperm from her womb, then she will urinate it out. Some normally use abortion to prevent pregnancy” (Male Adolescent—ADEZM_R9).

“If you want to prevent pregnancy after sleeping with a man and you discover that you are pregnant, there is a drug the nurse normally gives” (Female Adolescent—ADABF_R4).

### Beliefs and misconceptions about modes of action of contraceptive methods

Some adolescents were able to identify the modes of action of contraceptive methods while some were misinformed about their modes of action. Emergency pills were perceived to work by flushing away spermatozoa from a girl’s system after sexual intercourse, before fertilization takes place. Injectable hormonal contraceptives were perceived to work through blocking the uterus from getting impregnated. The following quotes highlight these misconceptions,

“….after having sex, the sperm that has been released by the male will wait for some hours before fertilization can take place. So after sex, she will take the pills and the pills will flush away the sperm” (Male Adolescent—ADAFM_10).

“For injections, if you inject the family planning drug, it will flow inside your body and go to block your womb so you cannot be impregnated by a man….” (Male Adolescent—ADEZM_R3).

With respect to condom, although most adolescents preferred it due to dual protection from pregnancy and STIs, male condoms were perceived to be potentially dangerous and life-threatening because they could break and enter into the body of the female sexual partner.

“…Condom is not good; when you use it, if care is not taken it will enter the body of the girl and it will make that person to die” (Female Adolescent—ADIKF_R10).

### Beliefs and misconceptions about condom use

Many adolescents appeared to know that, consistent use of condom during sexual intercourse protects, although not a hundred percent full proof, from unwanted pregnancy and STIs. However, there were some misconceptions about reusability of condom and effect of condom use on sexual pleasure during intercourse.

### Beliefs about reusability of condoms

Misconceptions about reusability of condoms were found to prevail among male adolescents compared to females. Some adolescent boys had the notion that particular brands of male condoms could be washed and reused for up to two or three times before disposal.

“The use of condom can prevent pregnancy and diseases. It can be washed and used two times” (Male Adolescent—ADIZM_R3).

“We have soft condom that will not harm you and the person you're having sex with. That is the one you can use two or three times; after using it you wash it” (Male Adolescent—ADIZM_R12).

### Beliefs about condom use and sexual pleasure

Notions about condom use and sexual pleasure appeared to vary for girls and boys. Some adolescent girls perceived that condom use during sex increases sexual pleasure because the assurance of being protected from STIs and pregnancy makes them better relaxed for pleasurable sex.

“The use of condom does not reduce pleasure, it improves pleasure because one feels protected.” (Female Adolescent—ADIZF_R6).

“Use of condom makes it pleasurable because one feels relaxed with it. The sex is enjoyable because both parties know they are safe from contracting disease and the girl getting pregnant” (Female Adolescent—ADABF_R2).

Adolescent boys had a different view from the girls. Although some of them recognized that condoms are useful for preventing pregnancy, they were of the opinion that it is more enjoyable having sexual intercourse without condoms because condoms interfere with the pleasure of direct ‘flesh to flesh’ contact.

“It (sex) is better flesh to flesh but the boy must be alert, he should make sure that he withdraws when he is about to release sperm." (Male Adolescent—ADAFM_R9).

“They (sexual partners) don't like using condoms because they won't enjoy it (sex).”(Male Adolescent—ADEZM_R13).

“It is very good to use flesh to flesh because using condom during sex might not be sweet, but it is advisable to use condom to avoid bringing shame to parents” (Male Adolescent—ADAFM_R7).

### Beliefs and misconceptions about contraceptive use and future fertility

Adolescents expressed their perceptions of how contraceptive use in adolescence affects fertility in future for both girls and boys. There was a general notion that contraceptive use in adolescence reduces fertility prospects for both sexes. Some were of the opinion that some girls who use contraceptives will be unable to get pregnant when they eventually get married and need to have children. Similarly, adolescent boys who use condoms were perceived to lose lots of spermatozoa and would be unable to impregnate women in future. For the above reasons, some respondents stated that adolescents should not use contraceptives. Some supporting quotes are highlighted below,

“It is not advisable for adolescents to use contraceptives because some women cannot get pregnant these days, and it is the type of contraceptive used in the past that led to their infertility” (Male Adolescent—ADAFM_R7).

"…Condom is not good because boys lose the sperm, and when they are mature and married and want to impregnate their wives it will not function again" (Female Adolescent—ADIKF_R10).

## Discussion

These findings indicate that although some adolescents had basic knowledge of methods of contraception, misconceptions still persist about types, modes of action and use of contraceptives. These findings underscore the need to provide adolescents with comprehensive and correct information on sexual and reproductive health through reliable sources such as schools, youth-friendly health centers and traditional/conventional media.

Their misconceptions about types of contraceptives reflect that there are gaps in knowledge which could be attributed to inaccurate sources of information. Adolescents mostly do not receive information from accurate sources such as experienced adults, parents and healthcare providers. Young people are most likely to receive and believe information they get through social media and friends as communication about SRH matters including contraceptives, barely occur in many African homes due to religious and cultural restrictions [6, 29]. This finding corresponds with a Nigerian study by Envuladu et al. which reported similar local/traditional methods used by women to prevent pregnancy [30]. Similarly, although majority of young people have basic knowledge about contraceptives, it has been reported that only a few have in-depth untainted knowledge about contraceptives [31].

Adolescents described the use of contraceptives in adolescence as a contributory factor to high rates of infertility among married couples. They opined that the use of contraceptives before adulthood among adolescents’ girls, blocks the womb of the female and increases her chance of not getting pregnant or delay pregnancy in marriage. In Pakistan and some Africa studies, misconceptions about the association of contraceptives with impotency in males, damage in females’ wombs and increased health problems have been reported among various categories of adolescents and young people [22, 32, 33]. On the contrary, ‘contraceptive’-related infertility has been attributed to the use of the various concoctions to prevent unwanted pregnancy, rather than proper use of modern contraceptive methods such as barrier methods and hormonal methods [34].

Perceptions about condom breakage and slippage into the female’s genitals during sexual intercourse as a factor that contributes to infertility and mortality resonated among adolescents. Although errors arising from condom use such as slippage during sex, late application or early removal have reportedly been identified in studies [35, 36], breakage and slippage during sexual intercourse rarely occur except when condoms are incorrectly used [37]. When condoms are incorrectly used only about 2% of them slip or break during sexual intercourse [37]. Among those who engage in sexual intercourse, consistent and correct use of condom remains the effective method of preventing most STIs like HIV [23].

Even though some adolescents had good knowledge about frequency of condom use and emphasized that it is not hundred percent full proof, some still had some misconceptions with respect to its reusability and effects on sexual pleasure. In describing condom use among sexual partnerships, the notion expressed by some adolescents is that condoms could be washed and reused, and this view was found to prevail among adolescent males. This corresponds with findings from a study among male college students where 1.4% of them reportedly reused condoms for sexual intercourse [38]. Errors arising from reuse of condoms have been repeatedly reported [35, 36]. Washing and reusing condom compromises its physical integrity/reliability and adolescents need to be educated about this. With respect to perceived effects of condoms on sexual pleasure, gender differences were also observed. Whereas adolescent boys opined that using condoms during sexual intercourse interferes with sexual pleasure, girls mentioned that it promotes sexual satisfaction and reduces the fear of unknown consequences of unprotected sexual intercourse. Correspondingly, another study conducted among male student population of a West African University revealed that approximately forty percent of respondents perceived condom use during sexual intercourse as interrupting sexual pleasure [26]. These notions about condoms interfering with sexual pleasure have been associated with low condom use and increased likelihood of adolescents to engage in unprotected sexual intercourse [39, 40]. The misconception that use of condom reduces sexual pleasure may perhaps be an important characteristic of masculinity which is worth exploring as this has been recognized in some previous studies [26, 41]. Addressing misconceptions about condom reusability should take center stage in SRH interventions targeting adolescent boys. There is also a need for in-depth exploration of how gender influences perceptions of sexual pleasure associated with barrier contraceptive methods.

A study limitation is the fact that qualitative research method is limited in its ability to produce generalizable findings. However, this study has highlighted various notions and misconceptions about contraceptives whose magnitude could be considered for quantitative measurement when designing questionnaires on sexual and reproductive health for adolescents. Given that misinformation could be easily spread among young people especially adolescents, through social media, peers in school and community, it would be important to estimate the magnitude of these notions among adolescents. Furthermore, due to the sensitive nature of our study, some participants may perhaps be uncomfortable disclosing complete information which leads to bias in response to explored questions. This study observed that engaging both in-school and out-of-school adolescents in same discussion limits full disclosure of information among either of the two schooling categories. Although there were no measures taken as regards to this during the study data collection, researchers are encouraged to have separate discussions with adolescents based on gender and schooling category. This is to ensure that they feel comfortable during discussion and reduce information bias in explored questions especially for sensitive topics. However, it is necessary that the present study findings are interpreted with some caution.

## Conclusion

Most adolescents appear to have some basic knowledge about types of contraceptives. However, this knowledge is tainted by misconceptions about types and modes of action of contraceptives, and reusability of condoms. Beliefs about effects of condoms on sexual pleasure were seen to vary by sex/gender. The persistence of misconceptions about contraceptives among adolescents raises concerns about the quality of information being disseminated about contraceptives and the potential influence of this on adolescents’ sexual behaviours and reproductive choices.

Concerted efforts should be made through educational and behaviour change interventions in schools and within communities to debunk persisting misconceptions about contraception, and properly educate adolescents on safe sex practices. This is to ensure that misconceptions are debunked and adolescents have access to correct and complete information for making healthy choices with respect to their sexual and reproductive behaviors. There is also a need to strengthen key messaging in media as well as engagement in more ‘misconception-busting’ information about contraceptives.

## Data Availability

Additional data from the research project could be made available by the corresponding author on reasonable request.

## Abbreviations

STI: Sexually Transmitted Infections
WHO: World Health Organization
IDRC: International Development Research Centre
IUD: Intrauterine Devices
FGDs: Focus Group Discussions
LGAs: Local Government Areas
